# Resolutions

**Published:** 1899-01

**Authors:** 


### Resolutions.

A the Tri-State Dental Meeting held at Put-in-Bay in June last, the following action was had:

Dr. J. R. Clayton, Indiana, presented the following resolution:

Whereas, Heeding the call of their country, a number of the younger members of our profession have enlisted in the army and navy of the United States; therefore, be it

*Resolved*, That we all regard with the highest admiration their patriotism and most cordially commend them for their unselfish devotion to their country and to the cause of humanity in thus voluntarily depriving themselves of the comforts of home, the society of family and friends, and the acquisition of the means for the enjoyment of the latter days of a useful and well-spent life.

*Resolved, further*, That we will watch the movements of their respective branches of the service with the greatest solicitude, well knowing that when they become engaged upon land or sea, that we will invoke the care of Him in whose hands are the destinies of nations, as well as individuals, that He may keep them over all of these sacrifices of health and life; that if it be His will, will carry them to the places made vacant by their leaving.

*Resolved*, That the Secretaries of the State Societies of Ohio, Indiana and Michigan be requested to transmit a copy of this resolution to each member of our profession now in the service of the United States.

